# Parameters of 150 temperate and boreal tree species and provenances for an individual-based forest landscape and disturbance model

**DOI:** 10.1016/j.dib.2024.110662

**Published:** 2024-06-26

**Authors:** Dominik Thom, Werner Rammer, Katharina Albrich, Kristin H. Braziunas, Laura Dobor, Christina Dollinger, Winslow D. Hansen, Brian J. Harvey, Tomáš Hlásny, Tyler J. Hoecker, Juha Honkaniemi, William S. Keeton, Yuta Kobayashi, Sofia Saenz Kruszka, Akira Mori, Jenna E. Morris, Stephen Peters-Collaer, Zak Ratajczak, Trond Simensen, Ilié Storms, Kureha F. Suzuki, Anthony R. Taylor, Monica G. Turner, Susan Willis, Rupert Seidl

**Affiliations:** a Ecosystem Dynamics and Forest Management Group, School of Life Sciences, Technical University of Munich, Hans‑Carl‑Von‑Carlowitz‑Platz 2, 85354 Freising, Germany; b Gund Institute for Environment, University of Vermont, 617 Main Street, Burlington, VT 05405, USA; c Natural Resources Institute Finland Luke, Latokartanonkaari 9, 00790 Helsinki, Finland; d Faculty of Forestry and Wood Sciences, Czech University of Life Sciences in Prague, Prague 6, Suchdol, Czech Republic; e Cary Institute of Ecosystem Studies, Box AB, Millbrook, NY 12578, USA; f School of Environmental and Forest Sciences, University of Washington, 3715W Stevens Way NE, Seattle, WA 98195, USA; g Vibrant Planet, PBC, Incline Village, NV, USA; h Rubenstein School of Environment and Natural Resources, University of Vermont, 81 Carrigan Drive, Burlington, VT 05405, USA; i Field Science Center, Tokyo University of Agriculture and Technology, 3-5-8, Saiwai-tyo, Fuchu, Tokyo, 183-8509, Japan; j Division of Biology, Kansas State University, Manhattan, KS 66506, USA; k Norwegian Institute for Nature Research, Torgarden, P.O. 5685, 7485 Trondheim, Norway; l Division of Forest, Nature and Landscape, Department of Earth and Environmental Sciences, KU Leuven; Celestijnenlaan 200E, 3001 Leuven, Belgium; m KU Leuven Plant Institute, KU Leuven, 3001 Leuven, Belgium; n Graduate School of Environment and Information Sciences, Yokohama National University, 79-7 Tokiwadai, Hodogaya, Yokohama, Kanagawa 240-8501, Japan; o University of New Brunswick, Faculty of Forestry and Environmental Management, 28 Dineen Dr, Fredericton, NB E3B 5A3, Canada; p Department of Integrative Biology, University of Wisconsin-Madison, Madison, WI 53706, USA; q Berchtesgaden National Park, Doktorberg 6, 83471 Berchtesgaden, Germany

**Keywords:** iLand, Simulation modeling, Species parameters, Wind parameters

## Abstract

Understanding the impacts of changing climate and disturbance regimes on forest ecosystems is greatly aided by the use of process-based models. Such models simulate processes based on first principles of ecology, which requires parameterization. Parameterization is an important step in model development and application, defining the characteristics of trees and their responses to the environment, i.e., their traits. For species-specific models, parameterization is usually done at the level of individual species. Parameterization is indispensable for accurately modeling demographic processes, including growth, mortality, and regeneration of trees, along with their intra- and inter-specific interactions. As it is time-demanding to compile the parameters required to simulate forest ecosystems in complex models, simulations are often restricted to the most common tree species, genera, or plant-functional types. Yet, as tree species composition might change in the future, it is important to account for a broad range of species and their individual responses to drivers of change explicitly in simulations. Thus, species-specific parameterization is a critical task for making accurate projections about future forest trajectories, yet species parameters often remain poorly documented in simulation studies.

We compiled and harmonized all existing tree species parameters available for the individual-based forest landscape and disturbance model (iLand). Since its first publication in 2012, iLand has been applied in 50 peer-reviewed publications across three continents throughout the Northern Hemisphere (i.e., Europe, North America, and Asia). The model operates at individual-tree level and simulates ecosystem processes at multiple spatial scales, making it a capable process-based model for studying forest change. However, the extensive number of processes and their interactions as well as the wide range of spatio-temporal scales considered in iLand require intensive parameterization, with tree species characterized by 66 unique parameters in the model. The database presented here includes parameters for 150 temperate and boreal tree species and provenances (i.e., regional variations). Excluding missing values, the database includes a total of 9,249 individual parameter entries. In addition, we provide parameters for the individual susceptibility of tree species to wind disturbance (five parameters) for a subset of 104 tree species and provenances (498 parameter entries). To guide further model parameterization efforts, we provide an estimate of uncertainty for each species based on how thoroughly simulations with the respective parameters were evaluated against independent data.

Our dataset aids the future parameterization and application of iLand, and sets a new standard in documenting parameters used in process-based forest simulations. This dataset will support model application in previously unstudied areas and can facilitate the investigation of new tree species being introduced to well-studied systems (e.g., simulating assisted migration in the context of rapid climate change). Given that many process-based models rely on similar underlying processes our harmonized parameter set will be of relevance beyond the iLand community. Our work could catalyze further research into improving the parameterization of process-based forest models, increasing the robustness of projections of climate change impacts and adaptation strategies.

Specifications Table

**Table:** 

Subject	Environmental Sciences: Ecological modeling
Specific subject area	Tree species parameters for process-based forest simulation
Type of data	Table, Database Raw, harmonized, partially tested
Data collection	Species parameters were compiled from previous published and unpublished studies performed by multiple research groups across Europe, North America, and Asia. Species parameters were initially derived from trait databases, the scientific literature (including peer-reviewed and grey literature), and forest inventories (e.g., National Forest Inventory data). Subsequently, parameters of multiple species and provenances were refined and evaluated against independent data and across multiple sites to ensure their robustness in application.
Data source location	Technical University of Munich, TUM School of Life Sciences, Ecosystem Dynamics and Forest Management Group
Data accessibility	Repository name: Mendeley Data Data identification number: 10.17632/58xdbwskp8.1 Direct URL to data: https://data.mendeley.com/datasets/58xdbwskp8/1
Related research article	Not applicable

## 1 Value of the Data

• Tree species parameters were obtained and harmonized (e.g., updating multiple versions of species parameters to the latest version) from research groups who have used the individual-based forest landscape and disturbance model (iLand) [1] across three continents and nine countries (Austria, Belgium, Canada, Czechia, Germany, Finland, Japan, Slovakia, and USA). The dataset [2] contains a total of 9249 entries for 66 parameters of 150 tree species and provenances from the temperate and boreal biomes. The parameters characterize the growth, survival (or mortality), and regeneration of trees within iLand as well as the simulated carbon and nitrogen dynamics.
• A second database [2] includes parameters addressing the susceptibility of trees to wind disturbance. This database includes a total of 498 entries for five parameters of 104 tree species and provenances.
• Tree species parameter sets were categorized into three uncertainty categories to indicate how thoroughly simulations of these species were evaluated against independent data. We identified 14 high confidence tree species parameter sets, 89 parameter sets with medium confidence, and 47 parameter sets with low confidence.
• The database facilitates the simulation of previously unstudied areas by providing a starting point for parameter testing and refinement. It furthermore allows the simulation of a wider set of tree species in existing study areas (e.g., to study assisted migration in the context of rapid climate change). Both databases presented here are ready to use in iLand. Since many parameters are relevant also in the context of other models the database has relevance for the forest modeling community.

## 2 Background

One important step in process-based modeling is to establish a set of parameters that characterize the simulated entities (here: trees), their responses to the environment, and their inter- and intra-specific interaction with other trees. Researchers have derived parameters for multiple species from various regions growing under a wide range of environmental conditions. They furthermore have evaluated simulations performed with these parameters against independent data sets characterizing specific aspects of the focal study system. By compiling and harmonizing the parameters from these different systems and sources, we synthesize the currently available work on characterizing temperate and boreal tree species in iLand, with the aim to improve model parameter reusability within the community, and to facilitate future model parameterization and application.

## 3 Data Description

The data are available as tables within an SQLite database file [2]. SQLite is an open-source database compatible with iLand and analysis tools like R [3]. The first table (“species”) encompasses all species parameters used in iLand for simulating demographic processes and environmental responses as well as carbon and nitrogen cycling. The second table (“wind”) specifically focuses on parameters defining the response of trees to wind disturbance. The structure of both tables is described in Tables 1 and 2, respectively.

**Table 1:** Names, descriptions and examples of tree species parameters used in iLand to characterize trees and simulate their demographic processes, environmental response, as well as carbon and nitrogen dynamics. Each row refers to a species-specific parameter in the SQLite database (Table species). For details on the use of the parameters in the iLand model logic see the online model documentation at https://iland-model.org.

Parameter name	Description	Example
isConiferous	0 for broadleaved species, 1 for conifers.	1
isEvergreen	1 for wintergreen species.	0
specificLeafArea	Factor to calculate one-sided leaf area from foliage biomass (m² kg^−1^).	5
turnoverLeaf	Annual senescence of foliage.	0.2
turnoverRoot	Annual senescence factor for fine roots.	0.05
HDlow	Function defining the lower bound of height to diameter ratios (i.e., open-grown trees).	170*(1)*d^−0.5
HDhigh	Function defining the upper bound of height to diameter ratio (for trees under heavy competition for light).	(195.547*1.004*(−0.2396+1)*d^−0.2396)*1
woodDensity	Wood density of the stem (kg/m3) (used for calculating the tree volume).	430
formFactor	Taper factor of the stem (used for calculating the tree volume).	0.423
bmWoody_a	Parameter a of the allometric equation (a*dbh^b) for stem wood biomass.	0.29
bmWoody_b	Parameter b of the allometric equation (a*dbh^b) for stem wood biomass.	2.09
bmFoliage_a	Parameter a of the allometric equation (a*dbh^b) for foliage biomass.	0.095
bmFoliage_b	Parameter b of the allometric equation (a*dbh^b) for foliage biomass.	1.56
bmRoot_a	Parameter a of the allometric equation (a*dbh^b) for coarse root biomass.	0.004
bmRoot_b	Parameter b of the allometric equation (a*dbh^b) for coarse root biomass.	2.79
bmBranch_a	Parameter a of the allometric equation (a*dbh^b) for branch biomass.	0.022
bmBranch_b	Parameter b of the allometric equation (a*dbh^b) for branch biomass	2.3
finerootFoliageRatio	The size of the fine root pool is defined relative to the size of the foliage pool (functional balance) i.e., fineRoots = poolsize foliage * finerootFoliageRatio.	1
cnFoliage	C/N ratio of foliage.	75
cnFineroot	C/N ratio of fine roots.	40
cnWood	C/N ratio of woody tissues (branches, stem, coarse roots).	300
barkThickness	Factor to calculate thickness of the bark (indicator of fire resistance) (bark thickness in cm = dbh * barkThickness).	0.065
probIntrinsic	Probability of a tree to survive maximumAge years.	0.01
probStress	Factor b_s that determines the probability of death based on a stress index.	6
maximumAge	Indicates a maximum age (years) for a species. Note that trees can grow older than this value in the model. This parameter is only used to determine aging and mortality probability and is not a deterministic cut-off age.	600
maximumHeight	Indicates a maximum height (m) for a species. Note that trees can grow taller than this value in the model. This parameter is only used to determine aging and mortality probability and is not a deterministic cutoff height.	60
Aging	Function used to calculate the decline in production efficiency with age (physiological and/ or based on max. height growth).	1/(1 + (x/0.55)^2)
lightResponseClass	Determines shade tolerance / light-use efficiency, where 1=very light-demanding, and 5 is very shade tolerant.	3.4
respVpdExponent	Exponent in the calculation of growth response to vapor pressure deficit.	−0.5
respTempMin	Lower threshold temperature (°C) for tree growth.	−2
respTempMax	Optimum temperature (°C) for tree growth.	17
respNitrogenClass	Nitrogen response class. Value must be >=1 and <=3. 3= highly nitrogen-demanding, 1= efficient with low available nitrogen.	2.2
phenologyClass	Link to a phenology class. 0= evergreen coniferous, 1= deciduous broadleaved, 2= deciduous coniferous.	0
maxCanopyConductance	Maximum conductance of the canopy for water (m s^−1^). Used in the calculation of transpiration.	0.02
psiMin	Maximum soil water potential (MPa) that a species can access (i.e. a species' permanent wilting point).	−1.5
maturityYears	Minimum age (years) required for a tree to produce seeds.	30
seedYearInterval	Interval between seed (masting) years. Each year has a probability of 1/seedYearInterval that a year is a seed year.	5
nonSeedYearFraction	Fraction of the seed production in non-seed-years.	0.25
fecundity_m2	Seedlings produced and surviving the first weeks per m² canopy cover (n m^−2^).	100
seedKernel_as1	Dispersal kernel parameter (m). The shape parameter for wind / ballistic dispersal (1–1/e = ∼63 % of wind dispersal is between 0 and as1 meter).	100
seedKernel_as2	Dispersal kernel parameter (m). Shape parameter for zoochorous dispersal (∼63 % of zoochorous dispersals are below as2 meter).	0
seedKernel_ks0	Proportion of zoochorous dispersal.	0
serotinyFormula	Function that decides (probabilistic) if a tree is serotinous. The variable is the age of the tree, expected return is a number between 0 and 1.	0.05
serotinyFecundity	Multiplier that increases fecundity for post-fire seed rain of serotinous species.	30
estMinTemp	Absolute minimum temperature (°C) for seed survival.	−39
estChillRequirement	Number of required days since the end of the last vegetation period between −5°C and +5°C.	56
estGDDMin	Minimum threshold of growing degree days for seedling establishment (GDD must be >GDDMin and < GDDMax to allow establishment).	177
estGDDMax	Maximum threshold of growing degree days for seedling establishment (GDD must be >GDDMin and < GDDMax to allow establishment).	3261
estGDDBaseTemp	Base temperature (°C) for GDD calculation. GDD is the running sum of (mean daily temp – GDDBaseTemp) for all days with mean temp > GDDBaseTemp.	4.3
estBudBirstGDD	Required GDD before bud burst. Calculation is similar to GDD described above, except that the counter is reset when mean daily temp is below 0°C.	255
estFrostFreeDays	Required number of days without frost (daily minimum temperature > 0 °C) in the year.	65
estFrostTolerance	Frost tolerance parameter for frost events after bud burst.	0.5
estPsiMin	Minimum soil water potential for establishment (MPa); establishment probability is reduced linearly between estPsiMin (*p* = 0), and field capacity (*p* = 1, no limitation). Null or 0 disables soil water limitation.	0
estSOLthickness	Effect of thickness of the soil organic layer on establishment probability. Multiplier calculated as exp(-estSOLthickness * SOLdepthcm). Null or 0 disables effect.	0
sapHeightGrowthPotential	Function to calculate the maximum height (m) of the sapling for the next timestep.	44.7*(1-(1-(h/44.7)^(1/3))*exp(−0.0398))^3
sapMaxStressYears	Number of consecutive years a sapling can withstand stress. If stress exceeds this threshold, the sapling cohort dies.	3
sapStressThreshold	Defines threshold for stress. If height increment / potential height increment is below sapStressThreshold, the sapling is stressed.	0.1
sapHDSapling	Saplings in iLand have a fixed height-diameter ratio, sapHDSapling, which is used to derive a diameter from sapling height.	80
sapReferenceRatio	Scaling factor to link unconstrained sapling height growth (see sapHeightGrowthPotential) to optimal environmental conditions for adult trees.	1
sapReinekesR	Stem number estimates of regeneration cohorts (n ha^−1^) are derived follow an allometric relationship (Reinekes stem density index). sapReinekesR is the maximum stem number for a dbh of 25.4 cm.	1450
sapSproutGrowth	Multiplier for accelerated height growth of resprouted tree cohorts in the regeneration layer (Null or 0 disables sprouting).	2
browsingProbability	Annual probability (ratio) that saplings (up to 2 m height) are browsed by game and ungulates.	0.1
snagKSW	The annual rate at which the biomass of a snag decomposes. This rate depends on species and is modified by environmental conditions (i.e., temperature and moisture).	0.015
snagHalfLife	Half-life (years) used for calculation of transition probability from snag to downed woody debris.	10
snagKYL	The annual rate at which the biomass of litter decomposes. This rate depends on species and is modified by environmental conditions (i.e., temperature and moisture).	0.15
snagKYR	The annual rate at which the biomass of downed woody debris decomposes. This rate depends on species and is modified by environmental conditions (i.e., temperature and moisture).	0.0807

**Table 2:** Names, descriptions and examples of tree species parameters used in iLand to simulate the response of trees to wind disturbance. Each row refers to a species-specific parameter in the SQLite database (Table wind). For details on the use of the parameters in the iLand model logic see the online model documentation at https://iland-model.org.

Parameter name	Description	Example
CReg	Critical turning coefficient (Nm kg^−1^) derived from tree pulling experiments.	132.2
crownAreaFactor	Empirical factor for the crown shape (fraction of area of the projected crown shape compared to a rectangle).	0.778
crownLength	Crown length of the tree given as fraction of tree height.	0.618
MOR	Modules of rupture (MPa).	36
wetBiomassFactor	Conversion factor between dry and wet biomass (wet = dry*factor).	1.85

**Fig. 1:**
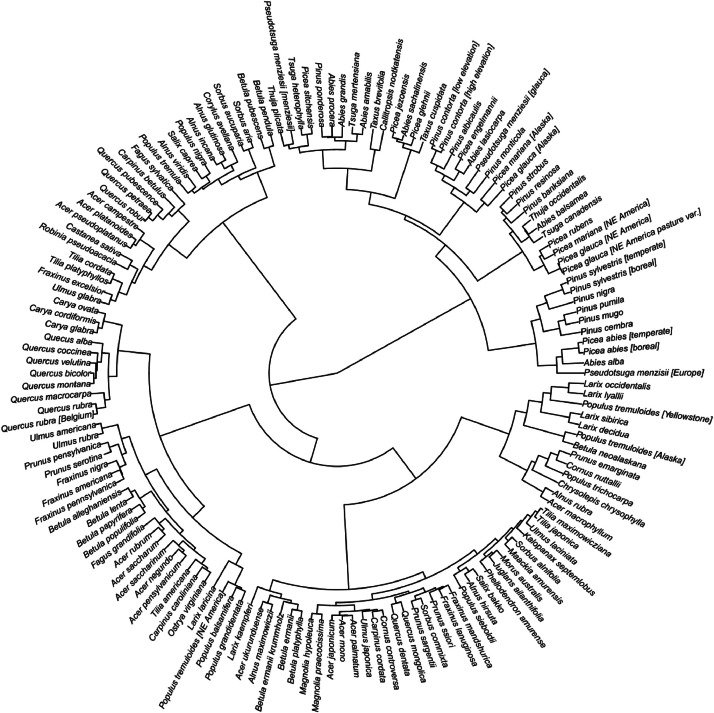
Parameter similarity among the tree species and provenances included in the dataset. The phylogram is based on an Agglomerative Hierarchical Clustering using a Gower distance matrix of 54 species parameters (i.e., those which could be meaningfully included in the analysis from the overall 66 parameters) for 150 tree species and provenances. The R code for the analysis can be accessed here: https://github.com/DominikThom/iLand-Species-Parameters.git.

The 150 species and provenances included in the database exhibit very different levels of similarity based on their species parameter values (Fig. 1). Broadleaved and coniferous tree species are clearly separated by their parameters, with few exceptions (i.e., deciduous conifers such as *Larix laricina* and *Larix kaempferi*). Moreover, clusters are clearly separated by continent. The most similar species are *Quercus robur* and *Quercus petraea*, whereas dissimilarity was highest between *Castanea sativa* and *Pinus contorta* (high elevation variety with serotinous cones).

## 4 Experimental Design, Materials and Methods

**Fig. 2:**
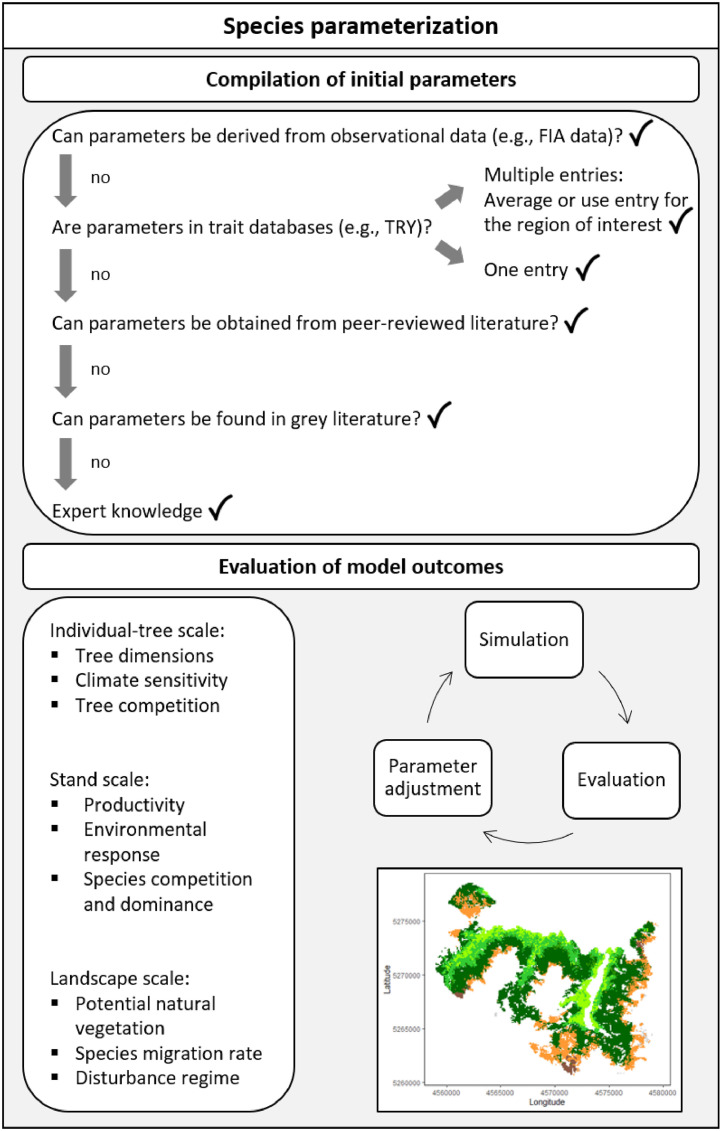
The steps to derive a robust species parameter set for process-based modeling. First, an initial parameter set is compiled from multiple sources. Subsequently, different patterns of ecosystems are simulated and evaluated against independent observations. Parameters might need to be iteratively adjusted (while ensuring that the parameter value remains within an ecologically plausible range), but local overfitting should be avoided to ensure realistic responses to novel environmental conditions.

The derivation of tree species parameters for process-based modeling is a time and resource intensive process that includes the compilation of an initial set of parameters (e.g., from the literature), followed by an iterative process of evaluation and refinement, ensuring that the parameters are consistent with the internal model logic, and that they reproduce the patterns expected for the simulated ecosystem [4] (Fig. 2). Here, we report parameters for the individual-based forest landscape and disturbance model (iLand) [1]. Introduced in 2012, iLand is an innovative process-based model for simulating the interactions among individual trees and their environment across a hierarchy of spatio-temporal scales, spanning from individual trees to the landscape and from minutes to millennia. iLand is based on first principles of ecology and is built around the representation of a multitude of ecosystem processes and their interactions. This process-based architecture enables robust projections of forest and disturbance dynamics also under changing environmental conditions. iLand has been successfully employed in temperate and boreal forests across Europe, North America, and Asia. For example, iLand has been used to simulate forest restoration in Asia [5], forest dynamics under climate change in Europe and North America [6,7] and disturbance regime shifts under climate change in Europe and North America [8,9] as well as changes in ecosystem services [10] and biodiversity [11] in Europe.

The parameters compiled here form the backbone of iLand simulation studies. They have been generated by the research community in a variety of ways and from numerous sources. We here briefly describe a default approach to estimating model parameters in the context of iLand, but acknowledge that the process can deviate substantially in individual cases as data availability for parameterization varies. Initial parameters are usually based on a combination of measurements, literature values, and expert estimates. Parameterization thus draws upon diverse data sources. We suggest to begin the parameterization by using observational data to derive species parameters (e.g., national forest inventories). Parameters that cannot be obtained from observational data might be found in species trait databases (e.g., the TRY database [12]). More parameters might be found in the (recent) peer-reviewed literature (e.g., [13]) or grey literature (e.g., [14]). If individual parameters are not available for a species of interest, expert knowledge (e.g., estimations based on the parameters of a closely ecologically related species) is frequently leveraged to fill gaps (see e.g. Fig. 1).

Initial parameters subsequently require careful refinement to ensure that they make up a coherent species parameter set that results in the emergence of realistic trajectories in the simulation. This refinement entails the thorough evaluation of the simulation results obtained with the respective parameters. Iteratively adjusting species parameters based on repeated analysis of model outputs and their comparison to independent data may be needed (see Fig. 2). We advocate for a pattern-oriented approach to model testing [4]. This involves comparing model outputs against both quantitative and qualitative information available for a study system. Given that iLand operates across multiple hierarchical scales, evaluation should also consider multiple scales. Depending on data availability, model evaluation focuses on:

- Individual-tree level:
  ○ *Tree dimensions* (e.g., average and distribution of diameter at breast height (dbh) and tree height) for each species. This is usually well documented from historical observations or can be obtained from old-growth forests.
  ○ *Climate sensitivity* (e.g., annual growth anomalies of trees). This can be obtained from regular measurements of tree growth (e.g., diameter increment from dendrometers).
  ○ *Tree competition* (e.g., growth response to tree neighbourhood). This can be evaluated against data from silvicultural trials (e.g., thinning or spacing experiments).
- Stand level:
  ○ *Stand productivity* (e.g., increment in: volume, basal area, dbh, and height). This can be tested for single-species stands and for stands with a mix of different species. Data for comparison can be obtained from local forest inventories and yield tables.
  ○ *Environmental responses* (e.g., changes in growth, mortality, and regeneration due to water stress). Data for comparison can, for instance, be derived from permanent forest monitoring plots or eddy covariance flux towers, but can also include the comparison of model behaviour across wide environmental gradients (e.g., across elevation).
  ○ *Species competition and dominance* (e.g., growth, mortality, and regeneration in species mixtures). Simulations can be compared with periodic inventories as well as species mixture trials from growth and yield studies.
- Landscape level:
  ○ *Potential natural vegetation* (i.e., the natural succession of species towards a tree species composition that is in dynamic equilibrium with the prevailing climatic conditions in the absence of human intervention). Simulations can be compared with local floristic assessments of forest types and expert estimates (e.g., gradients in species dominance across an elevational gradient), and can also use observations from unmanaged forests. The evaluation can focus on both the dynamic equilibrium species composition after a long simulation period but also the trajectory to this dynamic equilibrium, evaluating the simulated transition from early seral to late seral species over time.
  ○ *Species migration rate* (i.e., the movement of species across the landscape). Comparisons can be based on paleo records or terrestrial observations in response to ongoing climatic changes.
  ○ *Disturbance regime* (e.g., disturbance rates, sizes, frequencies, interactions etc.). Comparison of natural disturbance patterns and effects on the tree vegetation and subsequent regeneration can be performed based on remote sensing data, terrestrial inventories or other field data.

iLand is a process-based model based on first principles in ecology. Hence a site-specific adjustment of parameters is not recommended unless the performance of simulations in other regions increases simultaneously, as it could lead to local overfitting of parameters, reducing the robustness in applications under global change conditions. Rather, the parameters should broadly represent species in the simulation across a range of conditions, in some instances trading off precision for accuracy in simulated outcomes. For some species occurring under a very wide range of conditions, or for specific applications of the model, it is meaningful to distinguish individual tree species provenances in model parameterization (e.g., boreal vs. temperate *Pinus sylvestris*). The current dataset contains 21 provenances for nine tree species.

Most parameters compiled here underwent initial testing and evaluation (Fig. 2). However, the effort used and data available for evaluation varies considerably among species, and species are added and refined with the growing use of iLand. To communicate the resultant degrees of confidence in the parameterization of a tree species transparently, we assigned three categories (Table 3). Species parameter sets evaluated across a broad range of environmental conditions against diverse sets of independent data are classified as high confidence, those evaluated locally against limited data are rated as medium confidence, and those compiled but not evaluated are deemed low confidence.

**Table 3:** Confidence levels in the tree species parameters compiled here. Tree species parameter sets are categorized into high, medium, or low confidence. These confidence levels are primarily derived from the level of evaluations conducted for a species: Species evaluated across a broad range of environmental conditions against diverse sets of independent data are classified as high confidence. Species evaluated locally against limited data are rated as medium confidence, and species for which parameters have been compiled but have not been evaluated, yet, are deemed low confidence. Provenances indicated in square brackets.

## Limitations

• Only a few tree species and provenances contained in the database presented here have been thoroughly evaluated. The large majority of tree species and provenances parameters have moderate to low confidence and require further evaluation (Table 3).
• Parameters for rare species are frequently less robust due to fewer studies of species traits and limited independent data for evaluation (Table 3).
• Few provenances within species have been parameterized. Apart from these provenances, intra-specific variation in parameters is not considered in iLand.
• With the exception of regeneration parameters, average traits across a tree's life span are used within the simulation, although some traits may vary considerably with tree age.
• The tree traits reported here need to be interpreted within the context of the iLand model logic.
• Independent data is often lacking to thoroughly evaluate individual processes in the simulation and their underlying parameters.

## Ethics Statement

The authors have read and follow the ethical requirements for publication in Data in Brief and confirm that the current work does not involve human subjects, animal experiments, or any data collected from social media platforms.

## CRediT Author Statement

**Dominik Thom:** Conceptualization, Methodology, Data curation, Writing, Original draft preparation, Visualization. **Werner Rammer:** Conceptualization, Methodology, Data curation, Software, Writing. **Katharina Albrich:** Data curation, Writing- Reviewing and Editing. **Kristin Braziunas:** Data curation, Writing- Reviewing and Editing. **Laura Dobor:** Data curation, Writing- Reviewing and Editing. **Christina Dollinger:** Data curation, Writing- Reviewing and Editing. **Winslow Hansen:** Data curation, Writing- Reviewing and Editing. **Brian Harvey:** Data curation, Writing- Reviewing and Editing. **Tomáš Hlásny:** Data curation, Writing- Reviewing and Editing. **Tyler Hoecker:** Data curation, Writing- Reviewing and Editing. **Juha Honkaniemi:** Data curation, Writing- Reviewing and Editing. **William S. Keeton:** Data curation, Writing- Reviewing and Editing. **Yuta Kobayashi:** Data curation, Writing- Reviewing and Editing. **Sofia Saenz Kruszka:** Data curation, Writing- Reviewing and Editing. **Akira Mori:** Data curation, Writing- Reviewing and Editing. **Jenna Morris:** Data curation, Writing- Reviewing and Editing. **Stephen Peters-Collaer:** Data curation, Writing- Reviewing and Editing. **Zak Ratajczak:** Data curation, Writing- Reviewing and Editing. **Trond Simensen:** Data curation, Writing- Reviewing and Editing. **Ilié Storms:** Data curation, Writing- Reviewing and Editing. **Kureha F. Suzuki:** Data curation, Writing- Reviewing and Editing. **Anthony Taylor:** Data curation, Writing- Reviewing and Editing. **Monica G. Turner:** Data curation, Writing- Reviewing and Editing. **Susan Willis:** Data curation, Writing- Reviewing and Editing. **Rupert Seidl:** Conceptualization, Methodology, Data curation, Software, Writing.

## Data Availability

iLand Species Parameters (Original data) (Mendeley Data).
